# Heat Exposure Predicts Earlier Childhood Pubertal Initiation, Behavioral Problems, and Tobacco Use

**DOI:** 10.31586/gjeid.2025.1176

**Published:** 2025-01-16

**Authors:** Shervin Assari, Babak Najand, Hossein Zare

**Affiliations:** 1 Department of Internal Medicine, Charles R. Drew University of Medicine and Science, Los Angeles, CA, United States; 2 Department of Family Medicine, Charles R. Drew University of Medicine and Science, Los Angeles, CA, United States; 3 Department of Urban Public Health, Charles R. Drew University of Medicine and Science, Los Angeles, CA, United States; 4 Marginalization-Related Diminished Returns (MDRs) Center, Los Angeles, CA, United States; 5 Department of Health Policy and Management, Johns Hopkins Bloomberg School of Public Health, Baltimore, MD, United States; 6 School of Business, University of Maryland Global Campus (UMGC), Adelphi, MD, United States

**Keywords:** Extreme Heat, Climate Change, Substance Use, Tobacco Use, Behavioral Problems, Puberty, Child Development, Socioeconomic Status, Vulnerable Populations

## Abstract

**Background:**

Climate change has raised significant concerns about its impact on health, particularly for vulnerable populations such as children and adolescents. While extensive research has examined physical health effects, limited attention has been given to the influence of extreme heat on developmental and behavioral outcomes.

**Objectives:**

This study investigates the association between extreme heat exposure and early puberty initiation (ages 9–10), using data from the Adolescent Brain Cognitive Development (ABCD) study. It further explores how early puberty correlates with behavioral problems and tobacco use initiation.

**Methods:**

Data from 11,878 participants in the ABCD study were analyzed to examine the relationship between extreme heat exposure (independent variable) and puberty initiation (outcome). Behavioral problems and tobacco use initiation were evaluated as downstream outcomes of early puberty. Covariates included age, sex, and race/ethnicity, and behavioral problems were assessed using the Child Behavior Checklist (CBCL). Structural equation modeling (SEM) was employed for analysis.

**Results:**

Extreme heat exposure was significantly associated with earlier puberty initiation at ages 9–10. Early puberty, in turn, correlated with higher levels of behavioral problems and an increased likelihood of tobacco use initiation.

**Conclusions:**

These findings underscore the importance of addressing environmental factors such as extreme heat to reduce risks associated with early maturation, including behavioral and substance use challenges. Targeted interventions and policies are needed to mitigate the impact of extreme heat on child development, and longitudinal studies are essential to confirm these results and inform effective prevention strategies.

## 1. Introduction

Global temperatures have risen to unprecedented levels in recent decades, signaling profound climatic shifts since the mid-19th century. The Intergovernmental Panel on Climate Change (IPCC) [1] forecasts that the frequency and intensity of extreme heat events will continue to rise due to climate change. These patterns pose substantial challenges, particularly in economically disadvantaged regions where resources to address environmental stressors are scarce [2]. Extreme heat disrupts local economies by driving up operational costs, reducing productivity, and negatively affecting agricultural yields and consumer demand [3–6].

The health consequences of extreme heat are equally alarming [7, 8]. Heat exposure has been linked to increased mortality and morbidity rates, complications in pregnancy, and adverse mental health outcomes. Prolonged heat stress impairs physical and cognitive performance, elevates occupational health risks, and exacerbates pre-existing vulnerabilities [2]. Globally, nearly half of the population experiences high-heat episodes, with a significant proportion facing negative health impacts [2]. However, many of these risks are preventable through the implementation of strategic heat action plans and the adoption of behavioral and technological interventions [2]. Urban areas, in particular, face amplified heat-related challenges due to vehicular emissions, building heat waste, and limited green spaces, creating urban heat islands that exacerbate exposure [2].

Children are particularly sensitive to extreme heat. Research indicates that higher temperatures are strongly associated with increased emergency department visits among young children, particularly those aged 0–4. An increase of 13°F in maximum daily temperature has been linked to a 2.6% rise in emergency visits (95% CI: 2.2–3.0), with similar patterns observed across racial and ethnic groups and a range of health outcomes, including heat-related illnesses and injuries [9, 10]. For children living in poverty, the risks are even more pronounced. These children often lack access to mitigating resources such as air conditioning, increasing their exposure to heat-related stress and exacerbating inequalities.

Extreme heat can also influence developmental and behavioral outcomes, such as puberty initiation and high-risk behaviors like tobacco use. Emerging evidence suggests that socio-economic factors—including neighborhood SES, financial difficulties, and peer influences—may mediate the relationship between heat exposure and youth behaviors [26–29]. For example, Assari and Zare examined data from the Adolescent Brain Cognitive Development (ABCD) study, focusing on 11,878 children. Their findings revealed that higher levels of heat exposure were associated with lower SES, greater financial hardships, advanced pubertal development, and elevated delinquent behaviors. These results underscore the disproportionate impact of extreme heat on socioeconomically disadvantaged youth and highlight the pressing need for interventions to address these inequities [48].

### Objectives:

This study aims to fill a critical research gap by exploring the relationship between extreme heat exposure and puberty initiation at ages 9–10, using data from the ABCD study [16–25]. Additionally, it examines how early puberty correlates with behavioral problems and tobacco use initiation, reflecting the potential downstream effects of early maturation. By advancing understanding in this area, this research seeks to inform the development of effective interventions and strategies to mitigate the adverse impacts of climate change and extreme heat on child development.

## 2. Methods

### 2.1. Design and Sample

This study utilized secondary data from the Adolescent Brain Cognitive Development (ABCD) study [16–25], a large-scale, longitudinal research project involving a diverse sample of pre-adolescent children from various racial, ethnic, and socioeconomic backgrounds. The methodology for the ABCD study has been extensively detailed in previous publications. Key strengths of this dataset include its national coverage, longitudinal design, and diversity across race, socioeconomic status (SES), and geographical regions. Participants were primarily recruited through schools.

### 2.2. Analytical Sample

The analytical sample included all eligible youth from the ABCD study, irrespective of their racial, ethnic, or economic background. Participants were 9–10 years old at the baseline assessment, and a total of 11,878 children were included in this analysis.

### 2.3. Ethics

The ABCD study protocol was reviewed and approved by the Institutional Review Board (IRB) at the University of California, San Diego (UCSD). Written informed consent was obtained from parents, while children provided their assent to participate.

### 2.4. Study Variables

#### Puberty Stage at Age 9–10:

Puberty is marked by several key physical and growth changes that signal the development of secondary sexual characteristics. In females, this includes breast development and the onset of menarche (the first menstrual period), while in males, it involves genital development, an increase in testicular volume, and the deepening of the voice. Both genders experience the growth of pubic hair, the appearance of axillary (underarm) hair, and notable growth spurts characterized by rapid increases in height and weight. Changes in body composition also occur, with females typically experiencing an increase in fat distribution and males gaining more muscle mass. Males may also develop facial hair as part of these transformative physical changes. Together, these indicators reflect the complex biological processes of puberty and physical maturation. The Tanner Stages, also known as the Sexual Maturity Rating (SMR), is the most widely used scale for assessing pubertal development. This scale categorizes puberty into five stages, ranging from Stage 1 (prepubertal) to Stage 5 (full maturity), based on physical markers of development. In females, it evaluates breast development, while in males, it assesses genital development. Additionally, the Tanner Stages measure the growth of pubic hair in both genders, providing a comprehensive assessment of physical maturation. Alongside Tanner Stages, the Pubertal Development Scale (PDS) is another tool used to evaluate pubertal changes, offering self-reported or parent-reported insights into various aspects of puberty, including growth spurts, body hair development, skin changes, and other secondary sexual characteristics.

#### Race/Ethnicity:

Parent-reported data on the race and ethnicity of participants were categorized into non-Latino White (reference group), Black, Latino, Asian, and other racial/ethnic groups.

#### Family SES:

Socioeconomic status was assessed using indicators such as household income and parental education levels. Family income was a 1–10 interval measure, where a higher score indicated a higher income. The total combined family income in the past 12 months was asked. Responses were 1 = less than $5000; 2 = $5000; 3 = $12,000; 4 = $16,000; 5 = $25,000; 6 = $35,000; 7 = $50,000; 8 = $75,000; 9 = $100,000; and 10 = $200,000. Parents were asked, What is the highest grade or level of school you have completed or the highest degree you and your spouse / partner have received? Responses were 0 = Never attended/Kindergarten only; 1 = 1st grade; 2 = 2nd grade; 3 = 3rd grade; 4 = 4th grade 4; 5 = 5th grade; 6 = 6th grade 6; 7 = 7th grade 7; 8 = 8th grade; 9 = 9th grade; 10 = 10th grade 10; 11 = 11th grade; 12 = 12th grade; 13 = high school graduate; 14 = GED or equivalent diploma; 15 = some college; 16 = associate degree: occupational; 17 = associate degree: academic program; 18 = bachelor’s degree (ex. BA; 19 = master’s degree (ex. MA; 20 = professional school degree (ex. MD; 21 = doctoral degree. This variable was an interval measure with a range between 1 and 21. We adopted the Jaeger coding approach with a range from 31 to 46. For both of these variables, a higher score indicates higher SES.

#### Childhood Behavioral Problems:

Behavioral issues were measured using the Child Behavior Checklist (CBCL), where higher scores indicated greater behavioral challenges.

#### Tobacco Use Initiation:

Tobacco use initiation was defined as the first instance of reported use of any tobacco product, including cigarettes, e-cigarettes, and other related products, during any follow-up period.

#### Neighborhood Median Home Value:

This variable was derived from zip code-level data in the ABCD study’s residential history, representing the median home value in the neighborhood as a continuous measure of area-level SES.

### 2.5. Data Analysis

Data were analyzed using Stata software. Multivariable analyses employed structural equation modeling (SEM) to examine pathways linking heat wave exposure to tobacco use initiation. Mediators included childhood behavioral problems and MDD. Covariates such as age, sex, race/ethnicity, family SES, and neighborhood SES were included as potential confounders. Multicollinearity was checked and ruled out (all correlations were below 0.6). Results were reported as standardized path coefficients (beta), 95% confidence intervals (CI), and p-values.

## 3. Results

### 3.1. Puberty Initiation at Ages 9–10

**Figure 1.**
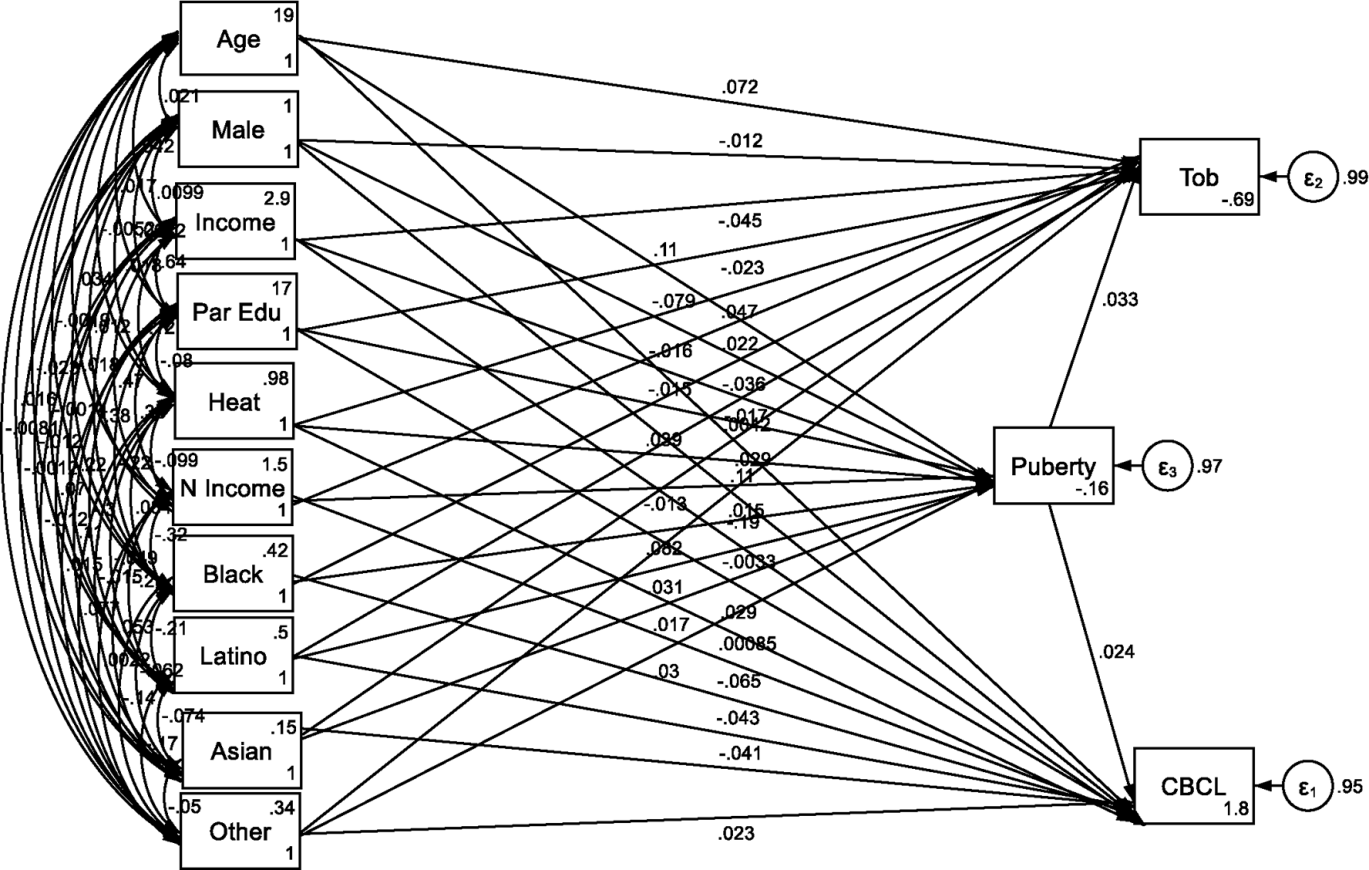
Summary of the Structural Equation Model (SEM) Note: Heat: Extreme Heat Exposure; N Income: Neighborhood Income; Other: Other Race; Par Edu: Parental Education; CBCL: Child Behavior Checklist Score; Tob: Tobacco Use Initiation.

Table 1 and Figure 1 summarize the SEM that tested factors associated with puberty initiation at ages 9–10. Extreme heat exposure was significantly linked to earlier pubertal stage (B = 0.039, SE = 0.011, 95% CI: 0.018–0.061, p < 0.001), suggesting a potential role of environmental stress in accelerating biological maturation. Age was positively associated with puberty initiation (B = 0.113, SE = 0.010, 95% CI: 0.093–0.132, p < 0.001), while male gender was negatively associated (B = −0.079, SE = 0.010, 95% CI: −0.099 to −0.059, p < 0.001).

Socioeconomic indicators, such as family income and education, did not show significant relationships with pubertal stage, nor did neighborhood income. However, race/ethnicity played a significant role, with Black (B = 0.082, SE = 0.012, 95% CI: 0.058–0.106, p < 0.001), Latino (B = 0.031, SE = 0.012, 95% CI: 0.008–0.054, p = 0.010), and “Other” race/ethnicity groups (B = 0.030, SE = 0.011, 95% CI: 0.008–0.051, p = 0.006) being positively associated with earlier puberty relative to the reference group.

### 3.2. Behavioral Problems

Puberty initiation at ages 9–10 was positively associated with behavioral problems (B = 0.024, SE = 0.010, 95% CI: 0.004–0.044, p = 0.017). Heat exposure also had a significant direct association with increased behavioral problems (B = 0.029, SE = 0.010, 95% CI: 0.010–0.048, p = 0.003). Male gender (B = 0.110, SE = 0.009, 95% CI: 0.092–0.127, p < 0.001) and lower family income (B = −0.189, SE = 0.013, 95% CI: −0.216 to −0.163, p < 0.001) were additional predictors of behavioral problems.

In contrast, Black (B = −0.065, SE = 0.011, 95% CI: −0.087 to −0.044, p < 0.001), Latino (B = −0.043, SE = 0.010, 95% CI: −0.063 to −0.022, p < 0.001), and Asian (B = −0.041, SE = 0.009, 95% CI: −0.059 to −0.024, p < 0.001) race/ethnicity groups were negatively associated with behavioral problems compared to the reference group.

### 3.3. Tobacco Use Initiation

Puberty initiation at ages 9–10 was positively associated with tobacco use initiation (B = 0.033, SE = 0.010, 95% CI: 0.014–0.053, p = 0.001), highlighting a link between early maturation and tobacco use initiation as a risk-taking behavior. Heat exposure also demonstrated a direct positive effect on tobacco use initiation (B = 0.047, SE = 0.010, 95% CI: 0.028–0.066, p < 0.001).

Other significant predictors of tobacco use included age (B = 0.072, SE = 0.009, 95% CI: 0.054–0.090, p < 0.001) and lower family income (B = −0.045, SE = 0.014, 95% CI: −0.073 to −0.018, p = 0.001). Black (B = −0.036, SE = 0.011, 95% CI: −0.058 to −0.014, p = 0.001) and Asian (B = −0.029, SE = 0.009, 95% CI: −0.048 to −0.011, p = 0.001) adolescents were less likely to initiate tobacco use compared to Whites.

## 4. Discussion

The primary goal of this study was to investigate the relationship between extreme heat exposure and early puberty initiation at ages 9–10, using data from the Adolescent Brain Cognitive Development (ABCD) study [16–25]. Additionally, we aimed to examine the socio-demographic factors—such as race/ethnicity, family and neighborhood socioeconomic status (SES), and financial hardships—that may amplify children’s vulnerability to extreme heat exposure. The study also explored how early puberty initiation correlates with subsequent behavioral problems and tobacco use, reflecting the potential consequences of early maturation.

Our findings identified a significant association between exposure to extreme heat and early puberty initiation at ages 9–10. Children exposed to higher levels of heat were more likely to come from low-SES families, live in economically disadvantaged neighborhoods, and face greater financial difficulties. Early puberty initiation, in turn, was linked to increased behavioral problems and tobacco use, emphasizing the broader developmental implications of early maturation associated with heat exposure.

Disproportionate exposure to extreme heat among Black youth can be attributed to structural and historical inequities. Systemic racism and historical segregation have concentrated Black communities in regions more prone to extreme heat, such as the southern United States, where poverty rates are also higher [30–34]. Urban heat island effects exacerbate these conditions, as Black families are more likely to reside in areas with limited green spaces, higher population density, and inadequate infrastructure [35–37]. These environmental stressors may contribute to physiological and psychological stress, potentially accelerating biological maturation and increasing the likelihood of early puberty.

Early puberty and pubertal initiation represent critical developmental milestones that often serve as gateways to profound changes in a child’s social environment, cognitive processes, and behavior. The onset of puberty introduces hormonal shifts that influence emotional regulation, decision-making, and risk perception, potentially heightening susceptibility to risk-taking behaviors such as tobacco use and substance experimentation. Socially, early-maturing youth may find themselves navigating environments that are more aligned with older peers, leading to increased exposure to peer influences and activities that may not align with their chronological age. These shifts often accelerate psychosocial transitions, including changes in identity, autonomy, and interpersonal relationships, which can create additional stress and challenge emotional regulation. Cognitive changes during this period, including greater impulsivity and heightened sensitivity to rewards, can further predispose early maturers to risk-taking behaviors and difficulties with behavioral control. The interaction of these social, cognitive, and biological changes underscores the importance of understanding early puberty as a dynamic and multifaceted gateway to developmental shifts, which may carry both opportunities and risks for long-term outcomes.

Children from lower SES families are similarly vulnerable. Limited access to resources, such as adequate cooling systems and well-insulated housing, increases exposure to heat stress [38–42]. Financial hardships further compound these risks, as families may lack the means to invest in cooling solutions or relocate to less heat-prone environments. Chronic stress resulting from financial insecurity can also disrupt hormonal regulation, potentially contributing to earlier puberty.

Neighborhood SES plays a critical role in amplifying vulnerability. Economically disadvantaged neighborhoods often lack infrastructure, such as parks, pools, or air-conditioned facilities, which can mitigate the effects of extreme heat [44]. These neighborhoods may also expose children to additional stressors, such as pollution and unsafe recreational spaces, further compounding the risk of early maturation and its associated behavioral outcomes.

The association between early puberty initiation and behavioral problems aligns with existing literature on early maturation. Physiological and hormonal changes linked to early puberty may lead to difficulties with emotional regulation, irritability, and impulsivity. These factors can increase the likelihood of engaging in risky behaviors, including tobacco use. Early puberty may also result in heightened social pressures and peer influences, further increasing susceptibility to delinquent behaviors and substance use.

### 4.1. Implications

These findings underscore the critical need for interventions to mitigate the impact of extreme heat on vulnerable populations. Strategies should include improving access to air conditioning, enhancing housing quality, and expanding urban greening initiatives to reduce heat exposure in low-SES neighborhoods. Additionally, schools and community organizations can play a pivotal role by offering structured activities in air-conditioned spaces, particularly during heatwaves. Public health campaigns should address the developmental and behavioral risks associated with early puberty, providing families with resources to support children during these critical transitions.

### 4.2. Future Research

Future studies should employ longitudinal designs to track the long-term impacts of extreme heat exposure on puberty timing and subsequent behavioral outcomes. Examining the cumulative effects of repeated heat exposure across critical developmental periods will provide a more nuanced understanding of how environmental stressors influence biological and behavioral trajectories. Further research should also investigate the interplay between extreme heat and other socio-environmental stressors, such as pollution and food insecurity, to create a comprehensive framework for addressing compound vulnerabilities.

Evaluating the effectiveness of mitigation strategies, such as community cooling centers, urban greening, and energy-efficient housing, is essential to inform public health and policy interventions. Research should also explore the role of educational institutions in promoting heat safety awareness and reducing exposure among children. Tailoring these interventions to account for regional and demographic differences will ensure equitable access to resources and improve outcomes for at-risk populations.

### 4.3. Limitations

This study has several limitations. Puberty initiation and subsequent behavioral outcomes were measured based on self-reported data, which may be subject to reporting biases. Future research should consider using biological markers or clinician assessments to validate self-reported puberty timing. Additionally, the cross-sectional nature of this analysis limits causal interpretations. Longitudinal studies are needed to clarify the temporal relationships between heat exposure, early puberty, and subsequent behavioral outcomes. The analysis may not fully account for all confounding variables, such as parental supervision, diet, and exposure to endocrine-disrupting chemicals, which could influence puberty timing. Expanding future models to include these variables would improve the robustness of the findings. Lastly, while the ABCD study provides a diverse sample, the results may not be generalizable to all geographic or demographic groups. Despite these limitations, the findings offer valuable insights into the impact of extreme heat on early maturation and its behavioral consequences.

## 5. Conclusion

This study highlights a significant association between extreme heat exposure and early puberty initiation at ages 9–10, with subsequent links to behavioral problems and tobacco use. Vulnerable populations, particularly Black youth and children from low-SES families and neighborhoods, face compounded risks due to environmental and socio-economic disparities. These findings underscore the urgency of targeted interventions to address the effects of extreme heat on child development and mitigate disparities exacerbated by climate change. Protecting vulnerable youth from these stressors is essential for safeguarding their health, development, and future well-being.

## Figures and Tables

**Table 1.** Summary of the Structural Equation Model (SEM).

Independent Variable		Dependent Variable	B	SE	95%	CI	p
							
Pubertal Stage (at Age 9–10)	→	Behavioral Problems	0.024	0.010	0.004	0.044	0.017
Age (9–10)	→	Behavioral Problems	−0.017	0.009	−0.034	0.001	0.066
Gender (Male)	→	Behavioral Problems	0.110	0.009	0.092	0.127	< 0.001
Family Income	→	Behavioral Problems	−0.189	0.013	−0.216	−0.163	< 0.001
Family Education	→	Behavioral Problems	−0.003	0.012	−0.027	0.021	0.784
Heat Exposure	→	Behavioral Problems	0.029	0.010	0.010	0.048	0.003
Neighborhood Income / 50000	→	Behavioral Problems	0.001	0.011	−0.020	0.022	0.936
Race/Ethnicity (Black)	→	Behavioral Problems	−0.065	0.011	−0.087	−0.044	< 0.001
Race/Ethnicity (Latino)	→	Behavioral Problems	−0.043	0.010	−0.063	−0.022	< 0.001
Race/Ethnicity (Asian)	→	Behavioral Problems	−0.041	0.009	−0.059	−0.024	< 0.001
Race/Ethnicity (Other)	→	Behavioral Problems	0.023	0.009	0.005	0.042	0.013
Intercept	→	Behavioral Problems	1.786	0.255	1.286	2.286	< 0.001
							
Pubertal Stage (at Age 9–10)		Tobacco Use Initiation	0.033	0.010	0.014	0.053	0.001
Age (9–10)	→	Tobacco Use Initiation	0.072	0.009	0.054	0.090	< 0.001
Gender (Male)	→	Tobacco Use Initiation	−0.012	0.009	−0.030	0.006	0.208
Family Income	→	Tobacco Use Initiation	−0.045	0.014	−0.073	−0.018	0.001
Parental Education	→	Tobacco Use Initiation	−0.023	0.012	−0.048	0.001	0.064
Heat Exposure	→	Tobacco Use Initiation	0.047	0.010	0.028	0.066	< 0.001
Neighborhood Income / 50000	→	Tobacco Use Initiation	0.022	0.011	0.001	0.043	0.043
Race/Ethnicity (Black)	→	Tobacco Use Initiation	−0.036	0.011	−0.058	−0.014	0.001
Race/Ethnicity (Latino)	→	Tobacco Use Initiation	0.004	0.011	−0.017	0.025	0.698
Race/Ethnicity (Asian)	→	Tobacco Use Initiation	−0.029	0.009	−0.048	−0.011	0.001
Race/Ethnicity (Other)	→	Tobacco Use Initiation	0.015	0.010	−0.004	0.034	0.111
Intercept	→	Tobacco Use Initiation	−0.693	0.261	−1.204	−0.181	0.008
							
Age (9–10)	→	Puberty Stage (Age 9–10)	0.113	0.010	0.093	0.132	< 0.001
Gender (Male)	→	Puberty Stage (Age 9–10)	−0.079	0.010	−0.099	−0.059	< 0.001
Family Income	→	Puberty Stage (Age 9–10)	−0.016	0.015	−0.046	0.014	0.291
Family Education	→	Puberty Stage (Age 9–10)	−0.015	0.014	−0.042	0.012	0.273
Heat Exposure	→	Puberty Stage (Age 9–10)	0.039	0.011	0.018	0.061	0.000
Neighborhood Income / 50000	→	Puberty Stage (Age 9–10)	−0.013	0.012	−0.037	0.010	0.270
Race/Ethnicity (Black)	→	Puberty Stage (Age 9–10)	0.082	0.012	0.058	0.106	< 0.001
Race/Ethnicity (Latino)	→	Puberty Stage (Age 9–10)	0.031	0.012	0.008	0.054	0.010
Race/Ethnicity (Asian)	→	Puberty Stage (Age 9–10)	0.017	0.010	−0.003	0.038	0.094
Race/Ethnicity (Other)	→	Puberty Stage (Age 9–10)	0.030	0.011	0.008	0.051	0.006
Intercept	→	Puberty Stage (Age 9–10)	−0.159	0.290	−0.728	0.410	0.583
